# Pseudoexfoliation Syndrome and Pseudoexfoliation Glaucoma: A Review of the Literature with Updates on Surgical Management

**DOI:** 10.1155/2015/370371

**Published:** 2015-10-29

**Authors:** Pasquale Plateroti, Andrea Maria Plateroti, Solmaz Abdolrahimzadeh, Gianluca Scuderi

**Affiliations:** ^1^Ophthalmology Unit, NESMOS Department, Sant'Andrea Hospital, University of Rome “Sapienza”, Via di Grottarossa 1035-1039, 00189 Rome, Italy; ^2^Ophthalmology Unit, DAI Head/Neck, Umberto I Polyclinic, University of Rome “Sapienza”, Viale del Policlinico 155, 00161 Rome, Italy

## Abstract

Pseudoexfoliation syndrome (PES) is a systemic disorder caused by progressive accumulation of extracellular material over various tissues. PES usually determines increased intraocular pressure, changes in the anatomical aspects of the optic nerve, and visual field alterations leading to the diagnosis of pseudoexfoliation glaucoma (PEG). Use of topical medical treatment usually leads to poor results in terms of long-term follow-up but many surgical techniques, such as Argon Laser or Selective Laser Trabeculoplasty, have been proposed for the management of PEG affected patients. The present paper is a review on the pseudoexfoliation syndrome and pseudoexfoliation glaucoma with an update on surgical management.

## 1. Introduction

Pseudoexfoliation syndrome (PES) is an age-related systemic microfibrillopathy, caused by progressive accumulation and gradual deposition of extracellular grey and white material over various tissues [1].

Presence of PES associated with elevated levels of intraocular pressure (IOP), related alterations on computerized perimetry examination, and/or changes in the anatomical aspects of the optic nerve determines the diagnosis of pseudoexfoliation glaucoma (PEG). Indeed, PES is considered as one of the most common causes of glaucoma.

Exfoliation syndrome was first described by Lindberg in 1917 who observed the presence of bluish-grey material deposited on the pupillary border in 50% of his patients with chronic glaucoma [2]. Vogt, in 1926, named the condition as “capsular glaucoma” since it was believed that the white flaky material could originate from peeling of the anterior capsule of the lens [3].

Several decades later, in 1954, the ocular pathologist Georgiana Dvorak-Theobald gave the term pseudoexfoliation syndrome to this disease, due to the observation of deposits of pseudoexfoliative material on the ciliary body, zonules, and lens capsule [4]. New theories now point to the minor role of the lens in the mechanism of the pathogenesis since pseudoexfoliative material has also been reported in pseudophakic and aphakic eyes [5].

Historically, it was thought that PES principally affected North European and, in particular, the Scandinavian population; thus, the international literature on the topic was scarce. Furthermore, PES associated glaucoma was treated in a similar manner to chronic open-angle glaucoma (POAG) [6, 7].

Although the epidemiology of PES varies widely and may depend on sex, age, and ethnic origin, it seems that the prevalence of this syndrome increases progressively among the following categories: people over age 50; ocular hypertensives; glaucoma patients; glaucoma patients admitted to hospital; glaucoma patients undergoing surgery; and patients blind from glaucoma or those with absolute glaucoma [8–10].

The scientific literature is still unclear on the mono- or bilateral involvement of the condition. On one hand, European reviewers describe a more frequent bilateral involvement of PES [11–13] while, on the other hand, American authors report a predominantly bilateral involvement [9, 14]. Interestingly, when the disease is clinically detectable in only one eye at slit lamp examination, conjunctival biopsy has revealed the presence of pseudoexfoliative material even in the fellow eye [15–17].

It has been reported that patients with bilateral PES tend to be older and have a higher incidence of glaucoma or ocular hypertension when compared to patients with unilateral involvement [18, 19].

Genetic factors are now considered as predisposing factors for PES, although results are not clear and studies are still ongoing [20].

PEG has been widely described as the result of the accumulation of pseudoexfoliative material, which obstructs the trabecular meshwork leading to an increase in IOP levels. As the awareness of PES has considerably increased, it has been widely demonstrated that PES can cause chronic open-angle glaucoma, but also angle-closure glaucoma, lens subluxation, blood-aqueous barrier impairment, and complications at the time of cataract extraction, such as capsular rupture, zonular dialysis, and vitreous loss [21].

Medical treatment usually leads to poor results, but several surgical procedures have been proposed to better cure the pathological manifestations of this syndrome [22–24].

The present paper is a review on PES and the clinical findings, diagnosis, and surgical management of the associated glaucoma and cataract.

## 2. Diagnosis and Clinical Findings

Within the eye, the fibrillar-granular pseudoexfoliation material characteristic of PES seems to be mostly produced from the lens capsule, ciliary body, corneal endothelium, zonules, and the iris. Electron microscopy and immunohistochemistry have led to the identification of the presence of extracellular matrix deposits also on other body tissues such as the liver, lung, kidney, gall bladder, and meninges [25–28]. The origin of this material is still unknown, but different enzymatic, histochemical, and immunological studies indicate that the fibrils and filaments are composed of noncollagenous proteins. Previous studies described similarities between this material and zonular elastic microfibrils, suggesting that pseudoexfoliation syndrome is a form of elastosis [29]. Furthermore, Scuderi et al. reported a case of PES associated with lattice corneal dystrophy, confirming the existence of a possible association of PES and amyloid accumulation [30].

Diagnosis of PES is based on the observation of pseudoexfoliative material on nearly all the structures of the anterior segment of the eye. Slit lamp examination, including gonioscopy and pupillary dilation, represents the gold-standard procedures for the clinical diagnosis of PEG. Poor and impaired pupillary dilation in PEG eyes seems to be caused by fibrillar deposits and ischemic damage to the iris causing stromal atrophy. An optimal mydriasis is necessary to observe the whole pattern distribution of pseudoexfoliative material over the anterior capsule of the lens.

Ultrasound biomicroscopy (UBM) can be helpful in those cases where alterations of the zonules and presence of iridodonesis or subluxation of the lens are suspected [31–33]. Iris fluorescein angiography can reveal the possible presence of iris ischemia [34, 35]. Since PEG is characterized by important fluctuations in diurnal IOP levels, intraocular pressure curve measurement is an important examination to monitor IOP levels at different times of the day in order to guide the clinician in the therapeutic management of patients [36].

### 2.1. Lens

PES is usually diagnosed by slit lamp examination that allows observing accumulation of whitish material deposits on the lens capsule. The typical bull's eye pattern disposition is probably due to the movement of the iris on the anterior lens surface, creating a double concentric ring aspect. Therefore, it is important to examine the anterior capsule of the lens after pupillary dilation, and in most cases the presence of three different areas is observed. The inner central disk-shaped zone, usually equivalent to the pupillary diameter, can be absent in almost 20% of cases. The intermediate clear zone, due to the iris movement on the anterior lens surface, and a more peripheral area containing radial striations have been demonstrated to be always present even in those cases where the central zone is absent or overlooked [3, 8, 37].

It has been observed that patients with PES present a higher percentage of nuclear cataract [38, 39]. Recent studies have also demonstrated a higher rate of subcapsular cataract in PES with respect to non-PES eyes [40]. Despite the widely reported higher incidence of cataract, its pathogenesis is still not clear. Cataract development seems to be related to the age of patients, although, in patients with unilateral PES, cataract appears to be more advanced on the affected rather than the unaffected eye [40].

### 2.2. Cornea

Slit lamp examination may demonstrate the presence of pseudoexfoliative material and pigment on the corneal endothelium that can be erroneously interpreted as inflammatory precipitates [41]. Confocal microscopy has demonstrated the presence of a lower number of endothelial cells in affected eyes and a consequent higher rate of guttae, which may probably be due to intermittent elevated levels of IOP [42–46]. The pigment observed on the corneal endothelium can sometimes be similar to the accumulation of pigment seen in the pigment dispersion syndrome [47].

Other nonspecific changes of the corneal endothelial cells include rarefaction and thinning of the cells, cytoplasmic vacuolization, phagocytosis of melanin granules, and abnormal extracellular matrix production [44].

### 2.3. Aqueous Humor and Anterior Chamber

Aqueous humor production in PES affected eyes has been demonstrated to be reduced [48] and associated with a disrupted blood-aqueous barrier with a consequent presence of higher levels of aqueous protein concentration [49], as well as sudden changes in levels of acid phosphatase [50], alpha1-lipoprotein and ceruloplasmin [51], cellular/plasma fibronectin [52], transferrin [53], alpha1-antitrypsin [54], and growth factors [55, 56]. Patients with PEG present greater serum concentrations of anti-*Helicobacter pylori* IgG-antibody (anti-HP IgG) compared to healthy patients [57] and an elevation of anti-HP IgG has been demonstrated in the aqueous humor of PEG and POAG patients [58].

### 2.4. Iris

Presence of pseudoexfoliative material is frequently observed on the anterior and posterior surface of the iris [59]. Irregular borders, due to the rubbing of iris against the lens and presence of grayish material deposits, characterize the aspect of pupil margin in PES [60, 61]. In most cases this is associated with poor or absent pupillary dilation as a result of atrophic and/or fibrotic changes in the iris sphincter muscle. Furthermore, the iris appears to be more rigid in patients with PES [62, 63].

Presence of deposits on both lens and iris is associated with more severe alterations in those patients with open-angle glaucoma [64].

Recent studies described various cases presenting iris ischemia and neovascularization, as a consequence of the deposition of pseudoexfoliative material on the vascular endothelium of the iris [34, 35].

### 2.5. Zonules and Ciliary Body

Weakness of the zonules is one of the main aspects of PES representing an important cause of complication during cataract surgery. It is thought that this zonular fragility can be caused by accumulation of pseudoexfoliative material on the ciliary processes and zonules, which may lead to phacodonesis [63, 65]. Schlötzer-Schrehardt and Naumann explain that the clinical instability of the zonular fibers is caused by histopathological alterations of the fibers and their altered anchorage in the defective basement membranes of ciliary body and lens [66].

### 2.6. Angle

Gonioscopy represents one of the fundamental examinations, which should be performed in patients with PES. Changes in both the aspect and depth of the angle commonly occur in PEG affected patients. Pigment and flecks of pseudoexfoliative material can be observed over the structures of the angle, especially along the Schwalbe line, where the pigment dispersion pattern is named “Sampaolesi's line” [67, 68].

## 3. Association of PES and Glaucoma

Pseudoexfoliative material can be observed in most cases on the pupillary margin and on the anterior lens capsule. PES is considered to be one of the most common causes of secondary open-angle glaucoma or ocular hypertension and early cataract development, because of its characteristics, including poor and impaired pupillary dilation, posterior synechiae, subluxation or dislocation of the lens and presence of weakened zonules [10]. It has been suggested that PEG may be due to the congestion of the trabecular meshwork [67]. Moreover, the prevalence of PES in glaucoma cohorts is significantly higher than in age-matched nonglaucomatous populations. Reported prevalence rates range from zero to 93%, with the highest rates in Scandinavia [69, 70].

PEG is mostly bilateral and asymmetric; if compared to POAG it presents a worse prognosis due to higher fluctuations in IOP levels and more severe optic nerve and visual field damage in affected eyes [71–77]. Furthermore patients with PEG usually present higher levels of IOP compared to those affected by POAG; moreover, various studies report a higher percentage of failure of medical management (prostaglandins, beta-blockers, adrenergic agonists, and carbonic anhydrase inhibitors) for PEG patients [22].

PEG increases with age and has a higher prevalence in patients between 60 and 70 years of age. Men are more affected than women [78], but this gender association is not always reproducible [79]. Although the prevalence in the general population varies from country to country, different studies describe a higher prevalence of PEG in Scandinavia [80, 81].

## 4. Management of Glaucoma and Cataract Surgery in PES Patients

The presence of pseudoexfoliative material in the anterior segment makes surgical procedures for both cataract and glaucoma more complicated.

Eyes with PEG respond poorly to medical therapy [22]. Topical drugs, such as latanoprost, travoprost, and dorzolamide-timolol combination, yield a good response in the first period of medical treatment, but PES is usually recalcitrant to glaucomatous medical therapy and this is the reason why patients affected by PES/PEG usually undergo laser or surgical therapy [23, 24].

### 4.1. Argon Laser Trabeculoplasty

In 1984 Tuulonen et al. reported that PEG affected eyes show a better response to Argon Laser Trabeculoplasty (ALT) with respect to POAG affected eyes [82, 83]. This outcome seems to be related to both diffuse pigmentation of the trabecular meshwork and the high baseline IOP values in eyes with PEG [84]. Moreover, according to Odberg and Sandvik ALT treatment may allow avoiding topical medical therapy up to 80% after 2 years and 67% after 5 years [85]. Apraclonidine Hydrochloride (Iopidine 0.5% and 1%, Alcon Laboratories, Inc.) has been reported to have a greater efficacy as temporary drugs in the prevention or reduction of IOP spikes after ALT [86].

### 4.2. Selective Laser Trabeculoplasty

Selective Laser Trabeculoplasty (SLT) can be considered as a repeatable procedure [87] and a good alternative to ALT. Although this method seems to be safe even in patients with POAG, the procedure still remains controversial [88, 89]. A recent study conducted by Goldenfeld et al. showed an important decrease of IOP up to 31.6% and a significant reduction of mean medications per patient in a 1-year follow-up [90, 91]. A lower success rate in SLT may be linked to the extent of angle treated in terms of degrees [91]. According to Nagar et al., a 360° SLT treatment has a greater efficacy than a 90° and 180° procedure [92, 93]. Song et al. also demonstrated a low efficacy and high failure risk of a 180° SLT [94]. A 360° SLT has been also performed in patients with POAG but results in terms of IOP were comparable to those obtained with monotherapy using topical latanoprost [92]. Baseline IOP can also influence final IOP decrease; in a study conducted by Shibata et al. on Japanese patients using a 360° SLT procedure, a baseline IOP > 21 mmHg led to a more significant decrease of IOP when compared to baseline IOP < 21 mmHg [95]. Later, Kent et al. compared ALT and SLT outcomes in patients with PEG and they obtained comparable results of IOP levels [96]. In terms of tolerability, SLT seems to have better results with respect to ALT [97]. Even if the incidence of IOP spikes may vary [90, 98], SLT seems to be less vulnerable when compared to ALT [85, 97].

### 4.3. Trabeculectomy

Trabeculectomy still represents the most frequent incisional procedure in the surgical management of PEG patients with advanced glaucomatous disease or when appropriate medical or laser treatment fails in controlling IOP levels [99]. It has been widely described that patients with PEG present a greater risk to develop surgical complications, due to the presence of zonular weakness or blood-ocular barrier dysfunction [100]. In a study conducted by Konstas et al., PEG patients who underwent trabeculectomy had a lower untreated postoperative IOP with respect to POAG patients. These results do not seem to be related to the duration of previous medical therapy [101]. More recent studies suggest similar outcomes of trabeculectomy in patients affected by PEG with respect to those affected by POAG in terms of IOP reduction, postoperative medical treatment, and surgical complications [102, 103]. Today, antifibrotic agents such as mitomycin C or 5-fluorouracil are commonly used to enhance the success rate of this procedure [99], similar to results obtained in POAG [104].

### 4.4. Angle-Based Procedures

The mechanism causing increase of IOP levels in PEG has been attributed to accumulation of pseudoexfoliative material and/or iris pigment in the trabecular meshwork. Therefore, surgical removal of this obstruction may lead to successful decrease of IOP. The angle-based procedures represent a group of techniques that seek to recover the natural aqueous outflow channels minimizing complications occurring in filtering surgical procedures, especially bleb-related issues [99].

The most commonly performed angle-based procedures are ab-interno trabeculectomy and trabecular aspiration. As nonpenetrating techniques, besides the reduction of postoperative complications after filtering surgery, these procedures have the advantage of preserving the conjunctiva so that penetrating surgery or aqueous shunt device implantation may be performed in the future [99].

Several studies analyzed the follow-up of these two procedures and according to Jacobi et al., trabecular aspiration tended to regress after 2 to 4 years of follow-up because of new accumulation of pseudoexfoliative material [105].

Ab-interno trabeculectomy, known as Trabectome (Neomedix Corp., Tustin, CA), consists in the ablation of trabecular meshwork from 60 to 120 degrees through the use of focused electrosurgical pulses associated with a continuous irrigation to avoid sudden deposition of pigmented and pseudoexfoliative material [99]. Ting et al. showed one-year results of Trabectome surgery in patients with POAG versus PEG. They demonstrated that IOP reduction and decrease in medication could be obtained in both groups after Trabectome treatment. Moreover a more significant rate of surgical success was obtained in the PEG group with 72.1% versus 62.9% of success achieved in POAG and PEG, respectively [106]. A study conducted by Klamann et al. compared these two procedures and found no differences in terms of IOP decrease, but patients with combined Trabectome and cataract surgery usually showed a stronger reduction of IOP levels [107]. Jordan et al. found similar IOP reduction in PEG patients who underwent Trabectome or combined Trabectome and cataract surgery [108].

Viscocanalostomy is considered another angle-based procedure, which avoids the risks associated with filtering surgery [109]. Carassa et al. compared viscocanalostomy against trabeculectomy in 50 patients. After 2 years of follow-up the success rates (IOP < 21 mmHg and no additional medication) were 76% for the viscocanalostomy and 80% for the trabeculectomy group. Furthermore when the IOP target was lowered to 16 mmHg they obtained a success rate of 56% and 72%, respectively [109].

Awadalla and Hassan evaluated combined cataract and viscocanalostomy surgery in PEG and POAG patients. A complete success rate with IOP values <21 mmHg and no glaucoma medication was obtained in 93.3% of PEG and 83.3% of POAG patients. Moreover, when the IOP target was lowered to <15 mmHg, the success rate was 83.3% in PEG and 53.3% in POAG patients [110].

The results of viscocanalostomy are encouraging especially in PEG patients; however, there is some reluctance regarding this technique because the final IOP target achieved is still not adequate for patients with advanced glaucoma [111].

### 4.5. ExPress Implant

The ExPress implant procedure (Alcon Laboratories Inc., Fort Worth, TX) was introduced to improve the trabeculectomy technique. These two procedures are quite similar but with the ExPress implant it has become possible to avoid iridectomy or sclerostomy since this implant is placed into the anterior chamber [99]. The learning curve for placing the ExPress implant is fast, especially for surgeons who are already skilled in trabeculectomy and this is why it is a common technique with more than 70000 performed procedures to date [112]. In a recent study conducted by Moisseiev et al., trabeculectomy and the ExPress technique were used and compared in patients with POAG, PEG, and “complex” glaucoma and no differences in terms of surgical success between these procedures were reported [113]. However, it has been reported that the ExPress implant technique is 3.5 times more expensive than trabeculectomy [114].

### 4.6. Aqueous Shunt Implantation

In 2012 a randomized controlled trial demonstrated a greater efficacy of aqueous shunt implantation versus surgical trabeculectomy [115]. 212 patients with IOL and/or failed filtering surgery were enrolled and randomly assigned to treatment with aqueous shunt or surgical trabeculectomy. Similar IOP values were described in the two groups but the failure probability and early and late onset complications were higher in the trabeculectomy with respect to the shunt implantation group [115, 116].

### 4.7. Cataract Surgery

In evaluating cataract surgery in patients with PES the control of IOP spikes and the degree of glaucoma should be taken into consideration in addition to corneal endotheliopathy, poor mydriasis, lens subluxation, and zonular instability [23]. An increased rate of complication during extracapsular cataract extraction in PEG eyes with respect to normal cataracts has been reported [117], although some authors found no differences [118]. Today phacoemulsification is the most frequent procedure performed in cases of pseudoexfoliative cataract and has a lower recurrence of complications [119, 120].

A good outcome of cataract procedures can be influenced by several key factors such as a good dilation and a wider capsulorhexis [23]. A study conducted by Scuderi et al. demonstrated that instillation of 10% phenylephrine and 0.5% tropicamide causes greater mydriasis than 2% ibopamine with a mean pupil diameter of 6.17 mm versus 5.33 mm, respectively, and a more significant dilation can be obtained through the combined use of both drugs (mean pupil diameter of 7.19 mm) [121]. Once good mechanical or pharmacological dilation is achieved, it is possible to reduce the stress on the capsular bag by creating a wider capsulorhexis which has been demonstrated to be helpful in the following steps of the surgical procedure [23]. In removing the lens, several techniques have been proposed to minimize stress on the weak PES zonules [122, 123]. Moreover, when choosing an intraocular implant, a 3-piece intraocular lens (IOL) is considered a better choice in patients with PEG [23]. However, zonular disintegration and capsular shrinkage in pseudophakic PEG eyes may lead to easier IOL luxation or dislocation into the vitreous [104] causing acute visual loss [124, 125]. Pars plan vitrectomy is considered the most suitable surgical approach in the removal of a dislocated IOL [126], although, in elderly patients who preserve good visual acuity, no procedure should be performed due to the increased risk of intraoperative and postoperative complications [104].

In addition to intraoperative complications, PES eyes have postoperative issues to be considered. This syndrome is usually associated with a higher risk of postoperative IOP spikes, iris vascular leak, and compromised blood-aqueous barrier [23, 127]. Following uncomplicated phacoemulsification there is an increase in average macular thickness as measured with optical coherence tomography which is not clinically significant; however, in a recent paper by Yuksel et al. the authors reported that patients with POAG and PEG had a greater increase of macular thickness with respect to controls [128, 129]. Yüksel et al. reported clinically significant cystoid macular edema with the same frequency in POAG, PEG, and controls. In these cases medical treatment can be a valid option. Although intravitreal steroids are efficacious in cystoid macular edema, this approach might not be appropriate in PEG due to the known effect of steroids on intraocular pressure [128, 130, 131].

Secondary cataract is also frequent and is usually due to some cortical remnants and weakened zonular support, which lead to migration of lens epithelial cells [104].

### 4.8. Combined Cataract and Glaucoma Surgery

A combined cataract and trabeculectomy procedure may be a good option in patients with PEG. Recently, Tran reported an approach with a new washout technique of pseudoexfoliative material in the iridocorneal angle or trabecular meshwork, which significantly decreases IOP and the amount of topical medical treatment required in the postsurgical period. This technique allows avoiding incisions or the need for sutures, thus, respecting the anatomical structures of the eye [119]. In 2005 Landa et al. reported similar results of this combined procedure in PEG versus POAG patients [120].

## 5. Conclusions

Several factors must be considered when evaluating patients with PES and/or PEG in order to determinate the most suitable management strategy. Careful examination and evaluation of all clinical aspects should be considered in order to choose the most appropriate medical and surgical approach for glaucoma and cataract surgery. Patients should be informed on the higher risk of possible complications.
